# The enigma of the near-symmetry of proteins: Domain swapping

**DOI:** 10.1371/journal.pone.0180030

**Published:** 2017-07-14

**Authors:** Maayan Bonjack-Shterengartz, David Avnir

**Affiliations:** Institute of Chemistry and the Lise Meitner Minerva Center for Computational Quantum Chemistry, The Hebrew University of Jerusalem, Jerusalem, Israel; International Centre for Genetic Engineering and Biotechnology, INDIA

## Abstract

The majority of proteins form oligomers which have rotational symmetry. Literature has suggested many functional advantages that the symmetric packing offers. Yet, despite these advantages, the vast majority of protein oligomers are only *nearly* symmetric. A key question in the field of proteins structure is therefore, if symmetry is so advantageous, why do oligomers settle for aggregates that do not maximize that structural property? The answer to that question is apparently multi-parametric, and involves distortions at the interaction zones of the monomer units of the oligomer in order to minimize the free energy, the dynamics of the protein, the effects of surroundings parameters, and the mechanism of oligomerization. The study of this problem is in its infancy: Only the first parameter has been explored so far. Here we focus on the last parameter–the mechanism of formation. To test this effect we have selected to focus on the domain swapping mechanism of oligomerization, by which oligomers form in a mechanism that swaps identical portions of monomeric units, resulting in an interwoven oligomer. We are using continuous symmetry measures to analyze in detail the oligomer formed by this mechanism, and found, that without exception, in all analyzed cases, perfect symmetry is given away, and we are able to identify that the main burden of distortion lies in the hinge regions that connect the swapped portions. We show that the continuous symmetry analysis method clearly identifies the hinge region of swapped domain proteins–considered to be a non-trivial task. We corroborate our conclusion about the central role of the hinge region in affecting the symmetry of the oligomers, by a special probability analysis developed particularly for that purpose.

## Introduction

The abundance of chiral rotational symmetry in protein oligomers[1–9] raises an interesting question: On one hand the list of advantages of this symmetrization is comprehensive and includes increasing the protein stability, avoiding excessive aggregation, enhancing of coding efficiency, reducing of synthetic errors, and inducing efficient cooperative regulation[1–5]. On the other hand, despite these advantages, we have shown recently[10] that perfect symmetry in proteins is rare: many oligomers which are built not only from similar (hetero-oligomers) building units but even from identical (homo-oligomers) deviate from ideal, perfect symmetry to some degree. This deviation is always detectable and measurable, and is beyond experimental uncertainty. What then is the origin of symmetry deviation that does not allow oligomers to maximize the symmetrization advantages? Recently we have proposed[10] that parameters which may be relevant for this question mark are: the minimization of the enthalpy of the interactions of the amino-acid units at the contact zones of the oligomeric subunits, which require giving away symmetry in order to attain that optimization (dealt with and proven in ref. [10]); relaxing the high entropic cost of maintaining perfect symmetry by increasing the number of possible microscopic conformations states of the protein; the operation of the property of any dynamic process that shifts objects away from symmetry; and the effects of the surrounding environment of the oligomer (solvent, crystal neighbors, the hydration shell), which may stabilize a distorted structure.

Here we explore the mechanism of the oligomerization as a potential source for symmetry deviation in protein oligomers. The rationale behind assuming that the formation of an oligomer may affect its symmetry is that the protein structure may reflect steps it underwent during its formation. For example, when the oligomer consists of at least three monomers, the mechanism of oligomerization is prone to be a sequential[11,12] (and not, at least in part, concerted), a route which may lead to de-symmetrization, because the first step is dimerization, and the next one is an interaction of a monomer with a dimer. In dimeric proteins—which are the main focus of this report—as well as in higher oligomers, the symmetry may be affected by the specific nascent stage after translation of all or part of the monomeric unit chains, at which association to form the dimer commences–it may take place either only after full completion of the monomer synthesis, or at an earlier stage[1,11–13].

**Fig 1:**
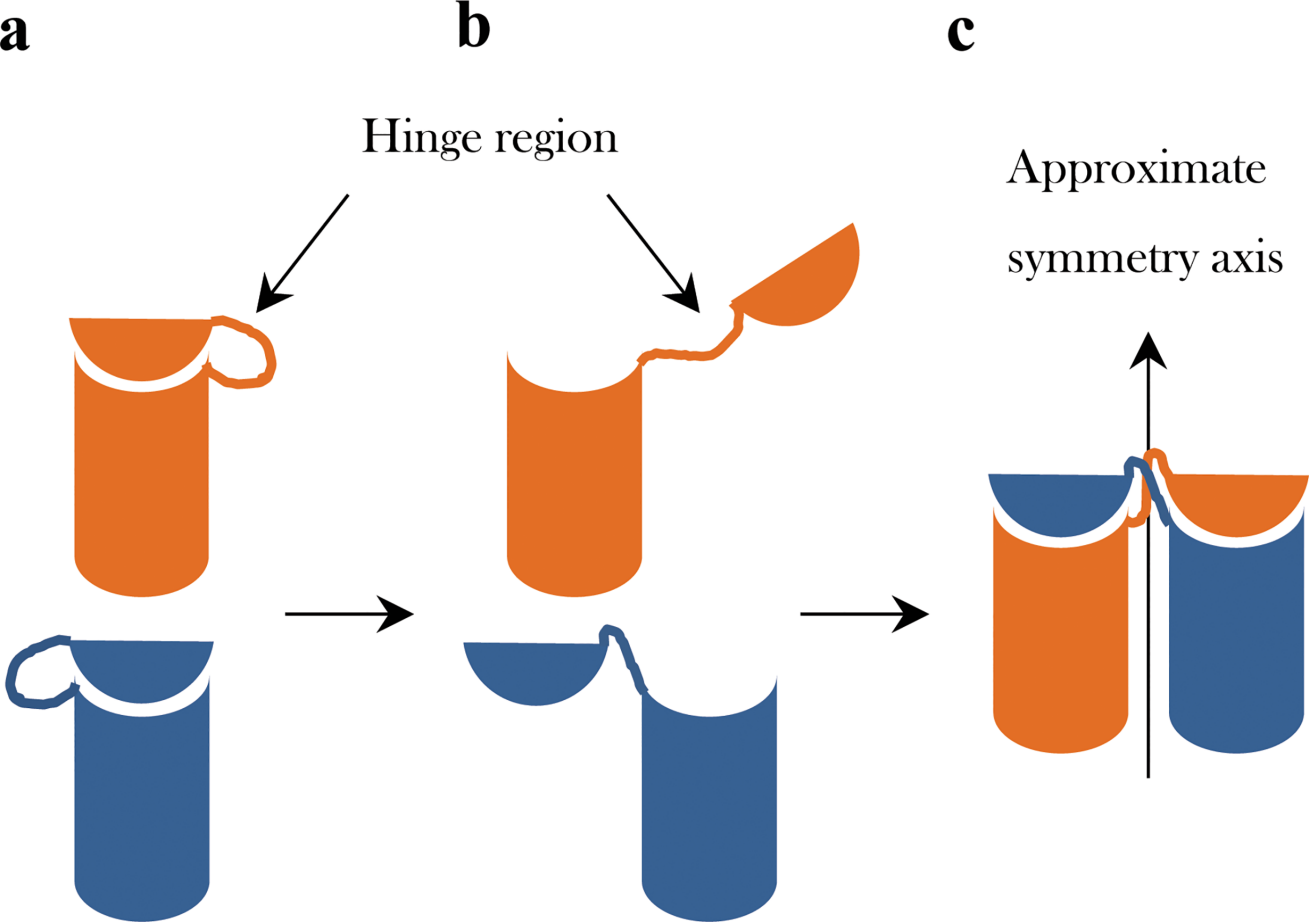
The domain swapping mechanism, demonstrated on the formation of a dimeric oligomer. (a) Two monomers with their folded potential hinge regions. (b) The monomers with their open hinge regions. (c) The dimerization, leading to the domain-swapped oligomer.

A particularly interesting mechanism of oligomerization which belongs to the latter option is domain swapping. The general idea of that proposed mechanism is that when two (or more) monomeric units assemble, they do so not by a simple aggregation process, but by aggregation that is accompanied by mixing or exchange of identical structural elements of the subunits[14–17]. In the swapping mechanism that mixing is carried out by exchanging (swapping) identical structural domains, so that two or more identical protein molecules form an intertwined oligomer, as shown in Fig 1. The resulting oligomer formed by this mechanism consists of subunits with the same structure as of the original monomer, except for the linking segments known as the *hinge regions* which connect the swapped domains (the secondary minor region) with the rest of the structure (the secondary major region). This oligomerization mechanism has been proposed for a wide range of proteins[15,18–24] where the size and nature of the swapped domains vary and may be as small as one secondary structural element or as large as a significant portion of the whole protein molecule. Likewise, the hinge region may be as small as consisting of three amino acids, but is it rarely larger than 15 amino-acids in length[21]. The majority of the oligomers formed by the swapping process display ***C***_***n***_ symmetry. This cyclic symmetry group contains a single axis of rotational symmetry, characterizing a protein with a quaternary structure of n subunits arranged in a ring, and which are related by an n-fold axis. The most prevalent ones are of ***C***_***2***_-symmetry[1] (which describes a half-turn symmetry), that is, dimers, which are therefore the focus of this report.

We report here our finding that, in agreement with our general observation cited above[10], that many dimers which are categorized as swapped-domain oligomers deviate from perfect symmetry. This observation has led us to investigate the hypothesis that the cause of this general symmetry deviation is related to the swapping mechanism, and particularly to the resulting linking hinges regions of the sub-units. This is so because the hinge region in each of the monomeric units is the only region that changes its secondary structure drastically when this mechanism operates: Often the change is from a folded minor-major region link within the monomeric state to an extended conformation link of these regions (Fig 1). If this is indeed the case then symmetry analysis which focuses on the symmetry relation of the two hinge regions (one in each subunit) may highlight them as carrying most or at least some of the distortive burden of these oligomers. In this report we show that, indeed, symmetry analysis identifies faithfully the hinge regions as significant symmetry distorted portions of the oligomers. It is also interesting to note in this context that in most cases of domain swapped proteins, the hinge region is located at or very close to the near-***C***_***2***_ axis (Fig 1).

We recall that supporting evidence for the swapping mechanism is not trivial, and that the full and detailed molecular swapping mechanism and its exact energetic aspects are still under development. From that point of view, the symmetry analysis presented below may also serve as supporting evidence for a swapping mechanism, when such is proposed. Propositions of domain swapping have been categorized as follows[15]: ‘*Bona fide* domain swapping’ proteins are such that their monomeric form is known; ‘Quasi-domain swapping’ proteins are such that a monomeric homologue is known; and ‘candidates for domain swapping’, which are proteins for which structural information of their monomer or monomeric homologue form is not available. In the last two decades several methods were developed[14,19–21,25–27] in order to address the question of whether a protein was formed by domain swapping mechanism and in order to identify the exact location and size of the hinge region in a protein oligomer suspected to be formed by that mechanism. The main method in this field was developed by Eisenberg and his co-workers[14] and is suitable for *bona-fide* domain swapping and quasi-domain swapping proteins, and utilizes a superimposability test between the hinge regions in the monomer and the dimer. See also instance 20 and 21 for more improved versions of Eisenberg's method. In cases of the third category—candidates of domain swapping proteins—the hinge loop region has been looked-after by several methods such as direct inspection of the protein’s crystallographic structure[19], or by the determination of the global minimum of the compactness profile of the oligomer[25]; of course, these methods are also suitable for the first two categories.

As was described above, tools for screening of domain swapped proteins already exist and the main contribution of the CSM analysis are for cases of uncertainty about the relevance of the domain swapping mechanism, for strengthening (or excluding) this proposed mechanism, and for accurately determining the protein hinge region. In the following sections, we first present the symmetry analysis that we developed in order to address proteins with proposed domain swapping mechanism; this method identifies the hinge region of swapped domain proteins with no need of structural information on the monomeric form of the non-swapped protein. We then provide an overall picture of the symmetry analysis results and their generality, include detailed investigation of several cases, and discuss the influence of the domain swapping mechanism on symmetry distortions of the whole oligomer, proving, we believe, that the formation of an oligomer may have profound effect on the resulting degree of symmetry.

## Methods

### The computational tools

The main focus of this study is the symmetry of proteins. The voluminous literature on this structural property of proteins has been limited by a qualitative descriptive language (“near-symmetry”, “approximate symmetry”, etc.)[1–4,7,28]. A quantitative approach which answers questions such as, ‘what is the degree of symmetry of an approximate-symmetry protein’, and, ‘by how much is one pair of hinges more or less ***C***_***2***_-distorted than another pair’ would allow to transfer the whole analysis and discussion to measurable facts. Thus, all of the symmetry analyses in this report are based on the Continuous Symmetry Measure (CSM)[29,30], a method for quantifying the degree of symmetry of a given object. According to the CSM approach, the ***G***-symmetry point group content of an object is the minimal distance between two objects: an original structure and a ***G***-symmetric structure, ${\bar{Q}}^{sym}$, which consists of the same atoms and connectivity and is the closest to the original distorted structure. This minimal distance of the object's vertices from the desired **G**-symmetry defined the measure *S(****G****)*:

$$S(G)=\frac{100}{d^{2}}\sum_{i=1}^{N}|{\bar{Q}}_{i}-{\bar{Q}}_{i}^{sym}{|}^{2},$$ (1)

where ${\bar{Q}}_{i}$ are the coordinates of the i^th^ atom of the original studied molecule, ${\bar{Q}}_{i}^{sym}$ are the coordinates of the i^th^ atom of the nearest structure which has the desired symmetry, the denominator is the root mean square size normalization factor of the original centered structure ($d=\sqrt{\sum_{i=1}^{N}{\left|{\bar{Q}}_{i}\right|}^{2}}$), and *N* is the number of analyzed atoms in the structure (see full details in[10,31]). It should be emphasized that this measure is inherently different than the rmsd analysis of the degree of similarity–the rmsd analysis does not evaluate the symmetry itself as a structural parameter, which is the key issue of this report. The range of the symmetry measure is 0 ≤ *S*(***G***) ≤ 1 and it is expanded by a factor of 100 for convenience (0 ≤ *S*(***G***) ≤ 100). If a structure is of perfect ***G***-symmetry, then *S*(***G***) = 0 and as the structure distorts from the perfect symmetry, *S(****G****)* increases. *S(****G****)* is a special distance function in that the nearest ${\bar{Q}}_{i}^{sym}$ is usually not known a-priori, but is determined by a minimization protocol described in detail in previous publications[29,32,33]. The measure is a global parameter, and therefore allows the comparison of various structures and various symmetries on the same scale. For alternative symmetry and chirality measures see, e.g., ref.'s [34] and [35].

In a previous study[10] we have introduced specific CSM computational tools for the evaluation of the symmetry content, *S(****G****)*, of proteins, two of which are relevant for this report: The "symmetry analysis of fragments" and the "local symmetry analysis". The fragments analysis, as the name implies, focuses on symmetry relations of specific portions of the protein structure. This analysis might reveal, on one hand, which regions in the protein carry the burden of the deviation, and on the other hand, which are barely deviating from perfect symmetry. The analyzed fragments can be as small as symmetry related atoms, but we found that the relevant minimal, useful unit in the context of proteins is the individual amino-acid; when this is used we refer to the analysis as a *local symmetry analysis*, which is a high-resolution tool: A CSM calculation is carried out on each pair of symmetry-matched amino acids within an oligomer, one amino-acid from each monomer. Each such calculation provides a local CSM values. This local symmetry analysis gives at a glance the relative deviations from symmetry within the oligomer structure, and specifically reveals which pairs of amino-acids are the most distorted ones in the structure. Detailed examples below, clarify it further.

### The analyzed proteins data

The selection of domain swapping protein structures for analysis was based on the datasets of Eisenberg[15] and of Huang[19] and on "3DSwap Knowledgebase of 3D domain swapping in proteins" database[36]. The coordinates of the analyzed proteins (${\bar{Q}}_{i}$ in Eq (1)) were taken from the crystallographic Protein Data Bank (PDB)[37]. All PDB entries in which the subunits are related by crystallographic symmetry are excluded from our data set. Therefore, we did not use any data in the database or in the literature mentioned above which was derived by placing only one sub-unit in the asymmetric unit and then assuming complete symmetry (these structures are by definition of *S(****G****)* = 0 value); the only crystallographic asymmetric units taken contain the full oligomer in the asymmetric unit.

## Results and discussion

### The CSM spectrum analysis

**Fig 2:**
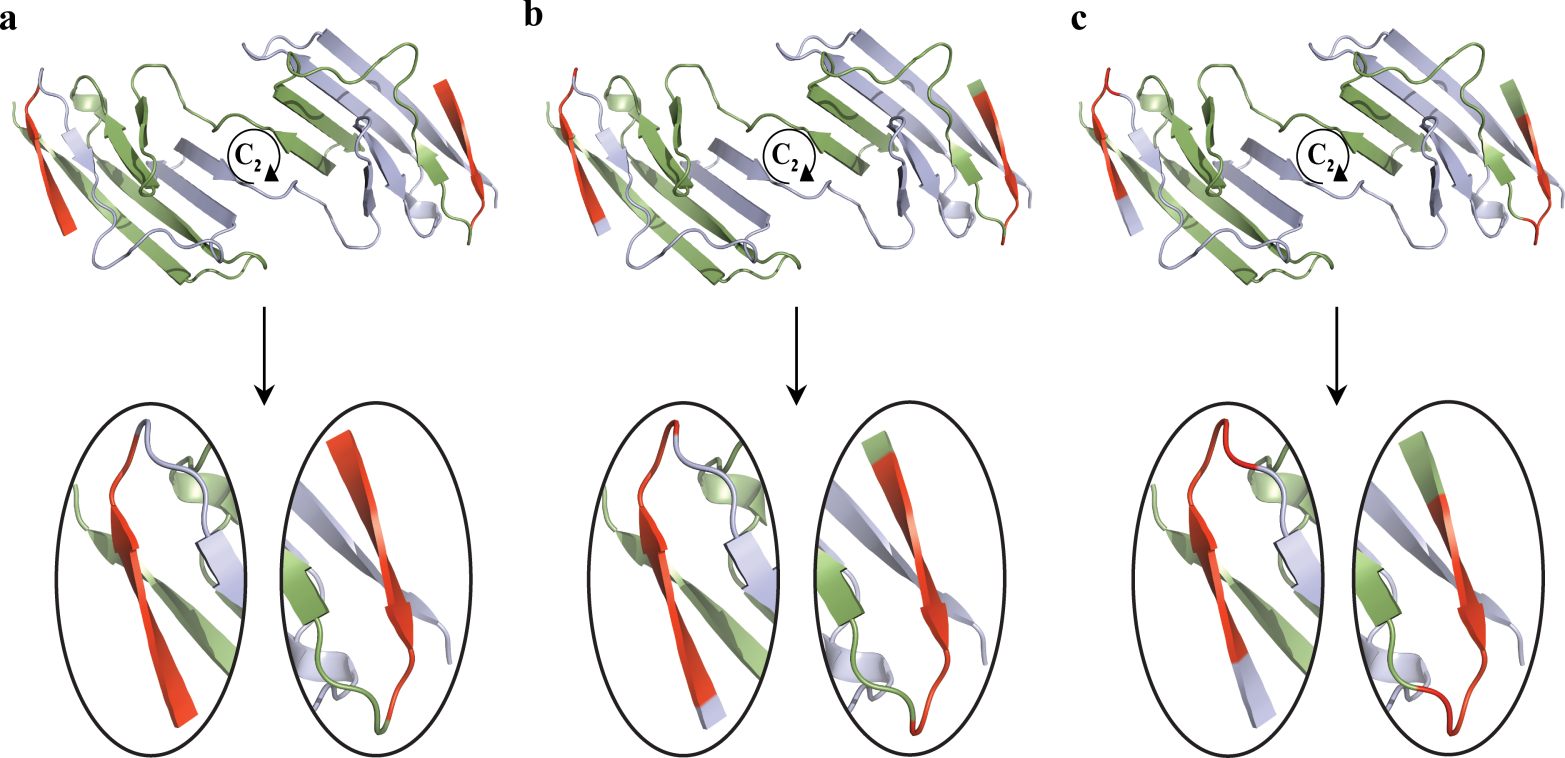
The running ruler method demonstrated on the engineered N-terminal domain of CD2 protein (PDB code: 1A64), starting from the N-terminal; running ruler size (red): seven amino-acids (h = 7). (a) The first segment, 1^st^-7^th^ amino acids segments-pairs. (b) The second segment, 2^nd^-8^th^ amino acids segments-pairs. (c) The third segment, 3^rd^ -9^th^ amino acids segments-pairs.

**Fig 3:**
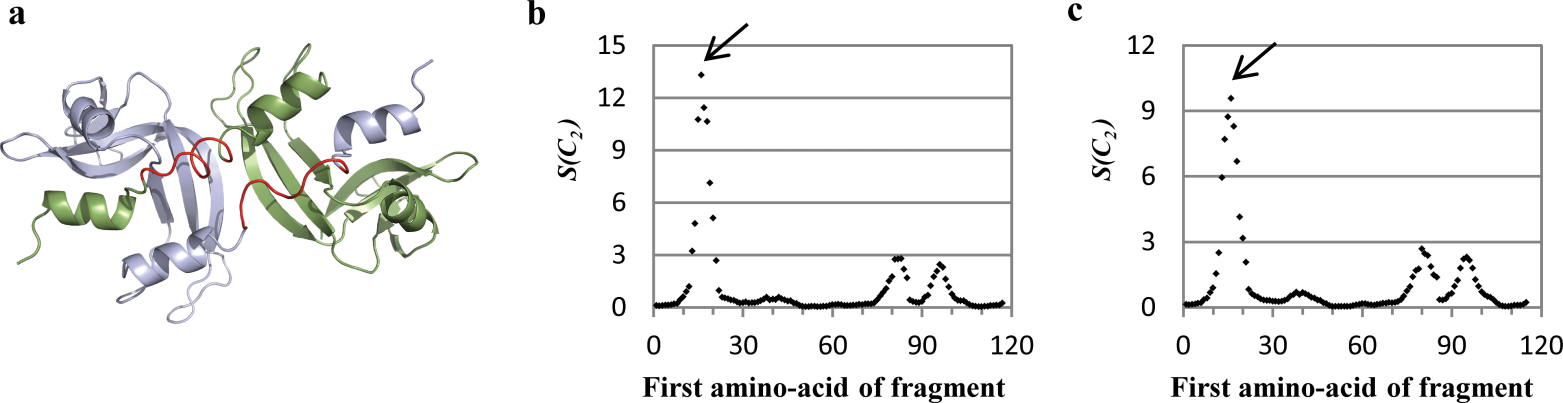
Running ruler symmetry analysis applied on RNase A N-terminal swapped dimer (PDB code: 1A2W). (a) Cartoon representation of the protein. Each subunit is indicated by a different color (blue and green), and the originally proposed hinge region is colored red. (b) CSM spectrum of the protein, the ruler size is as the length of the hinge region (8 amino acids). The black arrow indicates the hinge region. (c) CSM spectrum of the protein with a ruler size of 10 amino acids. The black arrow indicates the hinge region. For data source see ref. [38].

For the analysis of the rotational symmetry of the hinge regions–a pair of hinge regions in the case of ***C***_***2***_-symmetry—we developed the following extension of the symmetry analysis of fragments described above: A segment of *h* amino-acids is selected; *h* is defined as the *size of analysis ruler*. Then, (see Fig 2), starting with the 1^st^ amino-acid in the polypeptide chain of the monomer, the *S(****C***_***2***_*)* value of the first ***C***_***2***_-symmetry-related segment - 1^st^-*h*^th^ amino-acids segments-pair—is calculated (without H atoms), and a first CSM value is obtained. The ruler is moved then by a one amino-acid step to the second segment– 2^nd^-(*h*+1)^th^ amino-acids–and a second CSM value is calculated. The procedure is repeated one amino-acid after the other with the “running ruler” until (and including) the final segment of length *h* is reached. A total of *N* = *n* − *h* + 1 (where n is the number of amino-acids in the subunit) segments and their associated CSM values are obtained. A *CSM spectrum* is then plotted (Fig 3) in which the CSM value (*S(****C***_***2***_*)*) of the i-th segment (y-axis) is presented as a function of the position, n_i_, of the first amino acid in that segment (x-axis). The main idea is that zones in the protein which deviate more than their neighboring zones, should appear as peaks of high *S(****C***_***2***_*)* values. The running ruler can be of any size: As short as one amino-acid (*"local symmetry analysis"*), or as long as and the whole size of the polypeptide chain (*"all-atoms symmetry analysis of whole protein oligomer"* (see Ref. [10])). We have sampled different sizes of the ruler, and found that if nothing is known about the hinge in a suspected oligomer, one should use a ruler of size 10, and if a proposition exists about the size of a suspected hinge, one should test first that size as a running ruler (a case where we start with that proposed size, but then find a different size which is better will later be shown).

#### The generality of the symmetry distortion of the hinge range pairs

**Fig 4:**
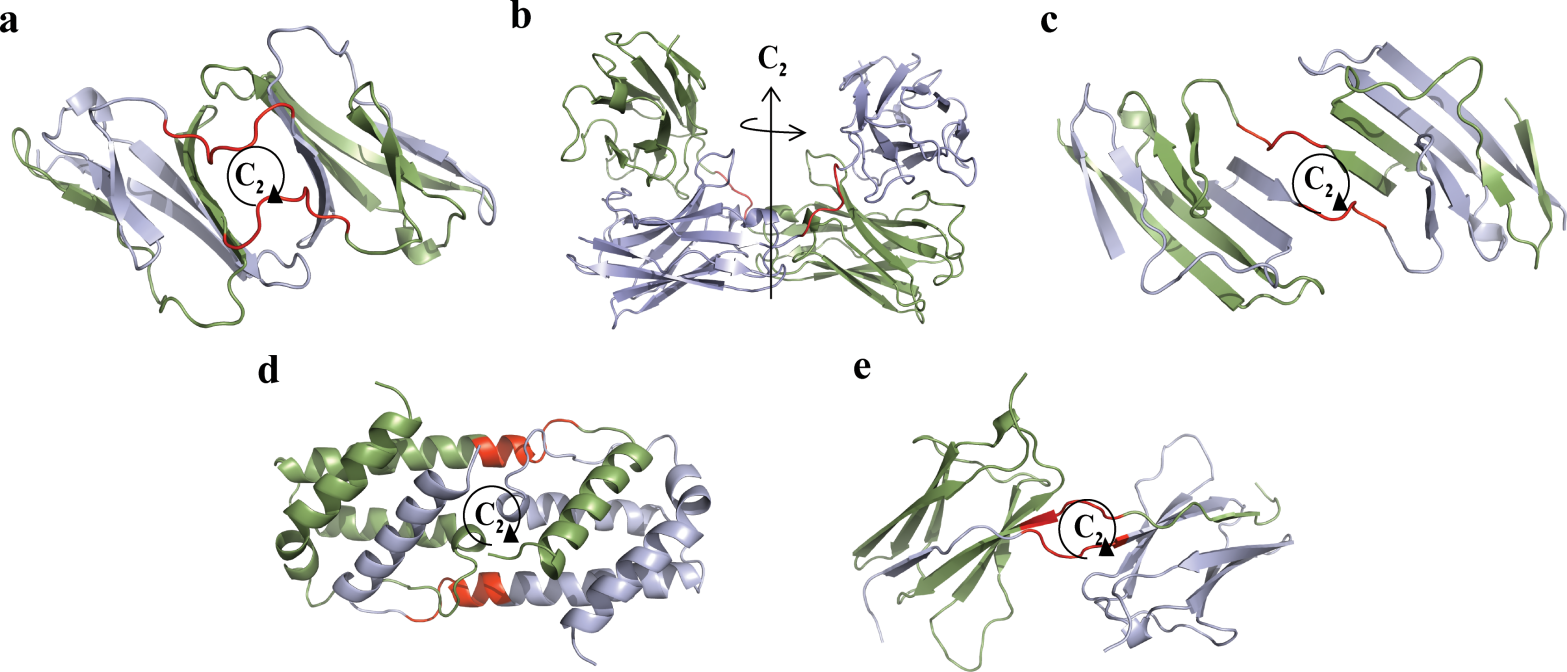
The proteins structures analyzed in Fig 5. Each subunit is indicated by a different color, and the originally proposed hinge region is colored red. (a) N-terminal domain of CD2 (PDB code: 1CDC), (b) Diabody (PDB code: 1LMK), (c) Engineered N-terminal domain of CD2 (PDB code: 1A64), (d) Interleukin-5 (IL-5, PDB code: 1HUL), (e) TrkA-d4 dimer (PDB code: 1WWA). For data sources see ref.'s [39–43].

**Fig 5:**
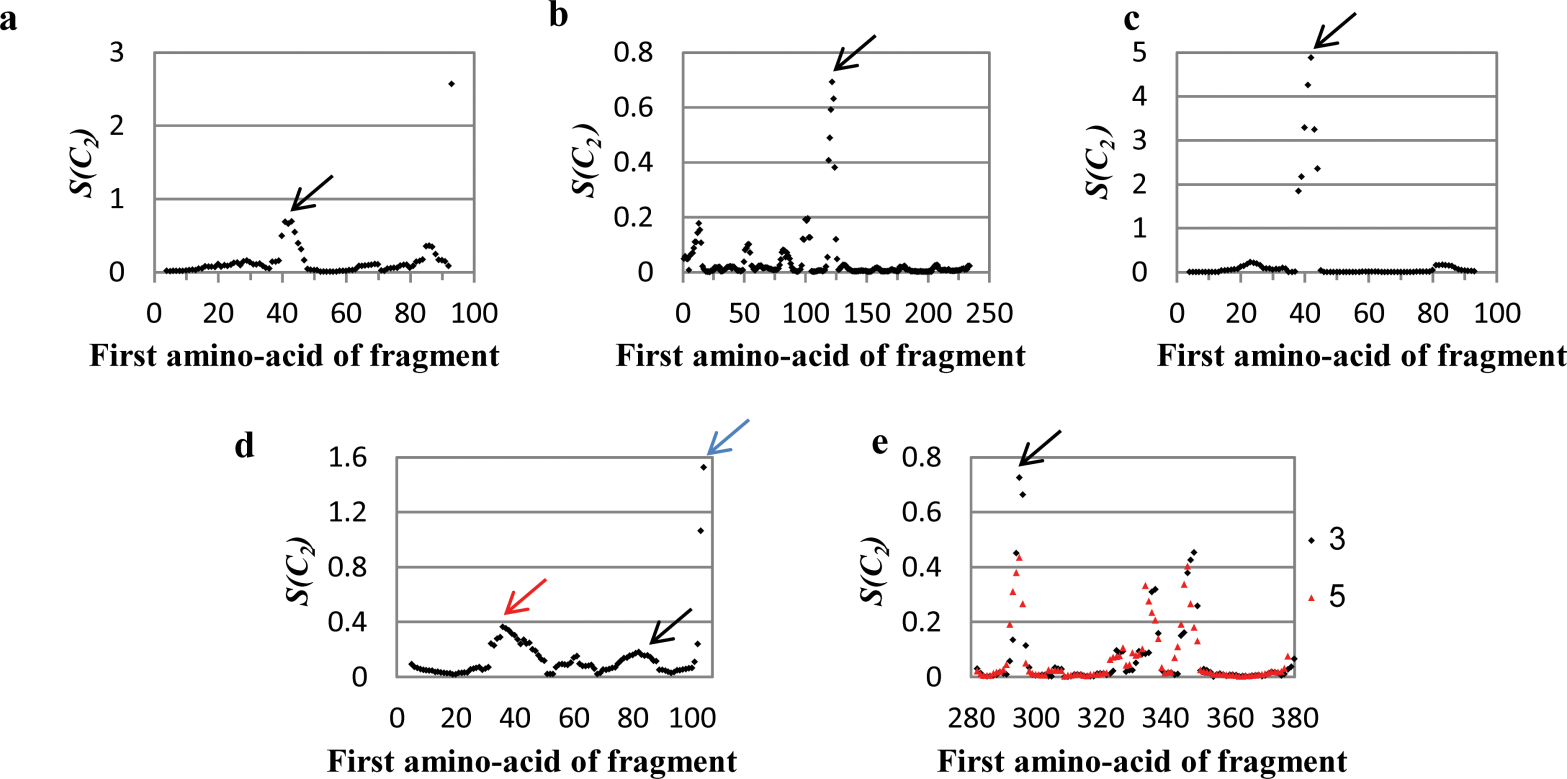
Running ruler symmetry analysis applied on proteins involved in 3D domain swapping. The black arrow indicates the hinge region; other colored arrows are explained in the text. (a) N-terminal domain of CD2, hinge region: 44–50, (b) Diabody, hinge region: 123–127, (c) Engineered N-terminal domain of CD2, hinge region: 44–50, (d) Interleukin-5 (IL-5), hinge region: 82–89, (e) TrkA-d4 dimer, hinge region: black– 297–299, red– 295–299. See Fig 4 for their PDB codes and cartoon representation and Table 1 for more information.

**Fig 6:**
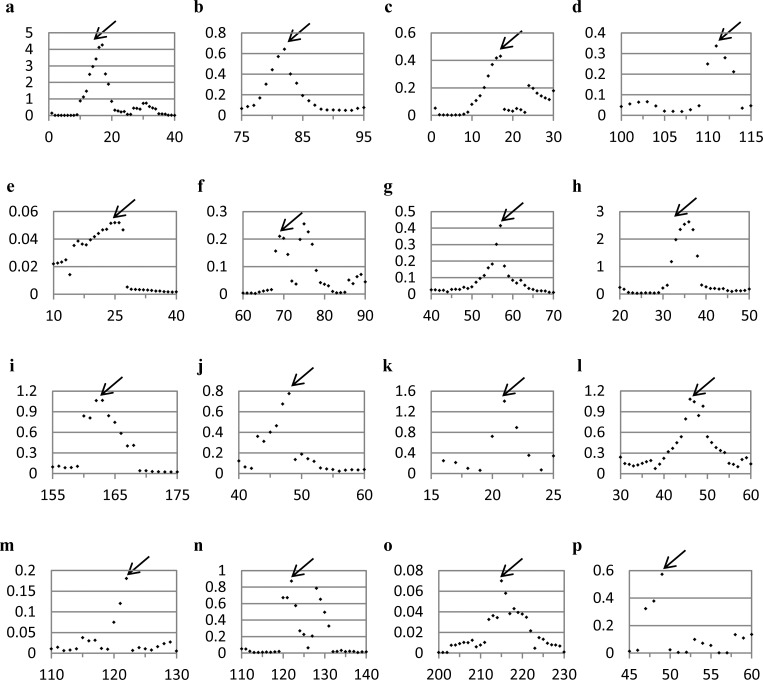
Additional running ruler symmetry analyses (see also S2 Fig); the neighborhood of the hinge region is shown. Black arrows—the hinge region. (a) Bovine seminal ribonuclease (PDB code: 1BSR), (b) β-crystallin (PDB code: 1BLB), (c) Human pancreatic ribonuclease chimera (PDB code: 1H8X), (d) RNase A N-terminal timer (PDB code: 1JS0), (e) Human glyoxalase I dimer (PDB code: 1BH5), (f) α-spectrin (PDB code: 2SPC), (g) Amyloid-like Cystatin C (PDB code: 1TIJ), (h) SH3 domain of Eps8 (PDB code: 1AOJ), (i) Circadian Clock Protein KaiA (PDB code: 1R8J), (j) Cyanovirin-N (PDB code: 1L5B), (k) Triggering receptor expressed on myeloid cells 1 (TREM-1) (PDB code: 1Q8M), (l) Cystatin A (PDB code: 1N9J), (m) Grb2-SH2 domain dimer (PDB code: 1FYR), (n) Odorant binding protein dimer (PDB code: 1OBP), (o) Cell division protein FtsZ (PDB code: 1W5F), (p) NrdH-redoxin (PDB code: 1R7H). See Table 1 for more information. For data sources see ref.'s [44–59].

We have carried out this CSM spectrum analysis on various protein structures suggested to be formed by a domain swapping mechanism. All-and-all, we have used 40 arbitrarily selected protein structures. For all proteins, the CSM spectra were obtained by the running-ruler method, and the spectra analyzed. A typical CSM spectrum is displayed in Fig 3B for RNase A N-terminal swapped dimer (PDB code: 1A2W), the structure of which is shown in Fig 3A. The general feature seen in Fig 3B is a sharp peak at the amino-acids positions range of 16–23, which very closely coincides with the amino-acids range originally suggested, namely, 15–22 –indicated in Fig 3A. This region is significantly more symmetry-distorted compared to any other segment in the protein, that is, it carries most of the burden of the symmetry. Let us assume that nothing is known about the hinge of this oligomer; we then have to use a ruler size of 10, which is displayed in Fig 3C. The CSM spectrum still identifies this region as the hinge region, but with less accuracy (the range now is 16–25). Table 1 summarizes similar observations made for proteins which belong to the *bona-fide* domain swapping and quasi-domain swapping categories, and that their hinge region locations were determined by Eisenberg et al.[15]; the related CSM spectra are collected in Figs 4–6. (in these spectra, one should consider the relative values of S(C2) in each spectra rather than its absolute values. It is seen from the Table that our method identifies hinge regions in all cases, and that in general they overlap well, with minor shifts of 1–2 residues, compared to the original propositions. Even the two last entries in Table 1 which display shifts of 3 and 4 amino acids, belong to large hinge regions, and represent overlaps of 7 and 9 amino-acids, respectively. *Without exception*, *in all proteins we analyzed*, *the hinge area appears as a peak*, even in the third category of “candidates of domain swapping proteins”—the generality is shown in Figs 5 and 6 and in S2 Fig. Thus, the formation route of the oligomer emerges as a key parameter in explaining its giving-up perfect symmetry. In the Probability analysis section we strengthen this conclusion with a statistical analysis, but some further comments on the data that can be elucidated from the CSM spectra is due first:

**Table 1:** Hinge region location.

PDB ID	Hinge region from the symmetry analysis	Literature hinge region[15]	Shift between the methods
1WWA	295–297	297–299	2
2SPC	70–73	72–75	2
1LMK	122–126	123–127	1
1JS0	111–114	112–115	1
1HUL	81–88	82–89	1
1CDC	43–49	44–50	1
1OBP	121–124	121–124	0
1H8X	17–24	16–23	-1
1FYR	122–124	121–123	-1
1A2W	16–23	15–22	-1
1AOJ	36–41	34–39	-2
1BSR	17–24	15–22	-2
1BLB	81–89	79–87	-2
1HT9	41–50	38–47	-3
1BH5	24–36	20–32	-4

#### Further comments on the CSM spectra

**Fig 7:**
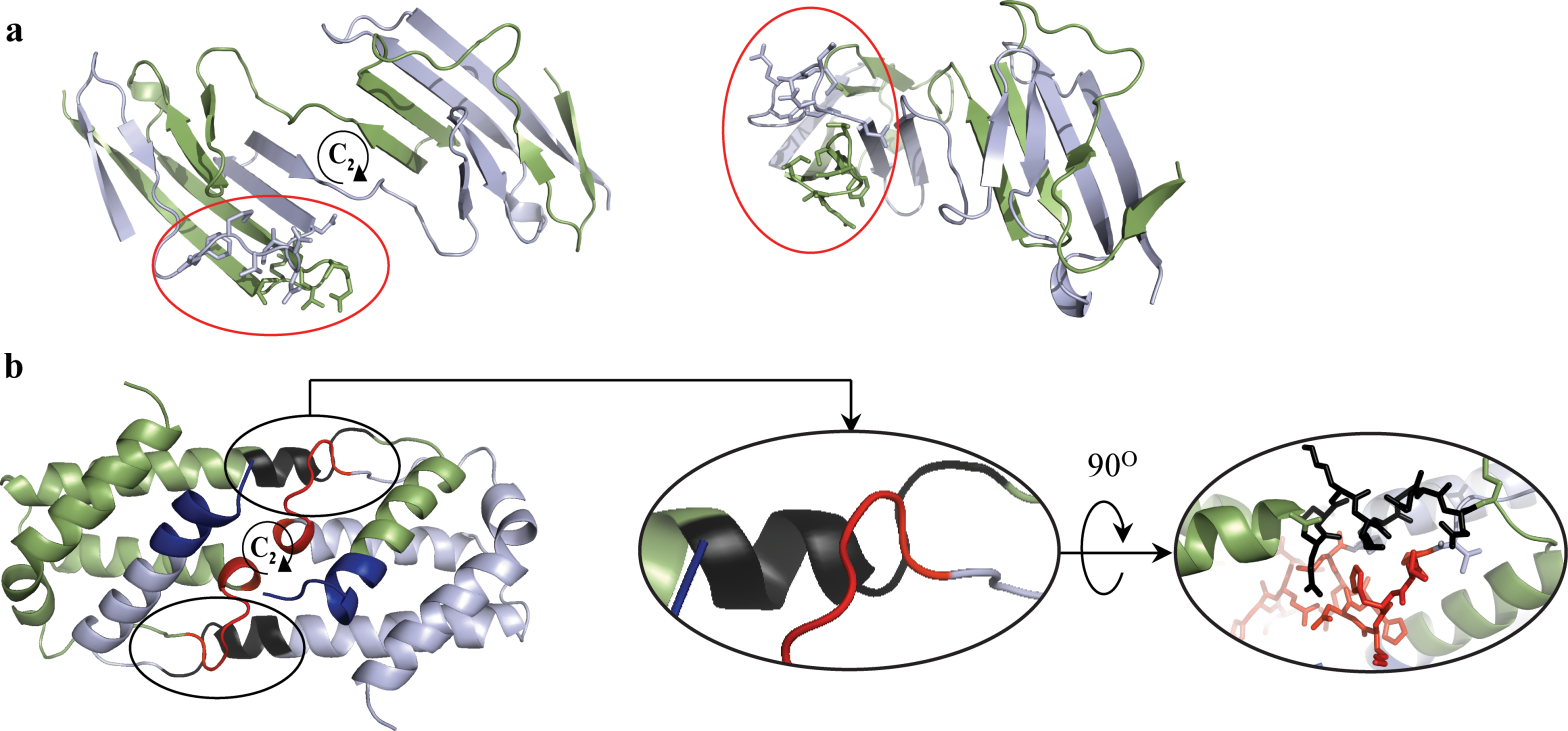
Focus on the origin of the symmetry distortion: each subunit is indicated by different color. (**a**) N-terminal domain of CD2 (PDB code: 1A64) from two different points of view. The amino-acids segments 21–28 and 81–88 are indicated by sticks representation. The interaction between those segments causes the symmetry distortion. These over-crowded regions are surrounded by red circles. (**b**) Interleukin-5 (PDB code: 1HUL). The regions which are indicated by colored arrows in Fig 5D are colored here respectively. The marked interaction area is seen from two different points of view. For data sources see ref.'s [41,42].

It is not necessary that the hinge region is the only portion of the protein which is ***C***_***2***_*-*symmetry distorted, or that the hinge pair is the most symmetry-distorted region in the oligomer. For instance, let us look again at the CSM spectrum of the engineered N-terminal domain of CD2 (Fig 5C), which has seven amino-acids hinge loop, located at the amino-acids 44–50. The most distorted region in the structure, as indicated in the spectrum indeed points to the hinge segment at the amino-acids 42–48, with minor shift of two residues compared to the originally suggested hinge region (44–50, according to 3DSwap Knowledgebase of 3D domain swapping in proteins)[36]. This region is significantly different from the rest of any segment in the protein, from the symmetry point of view: it carries most of the burden of the symmetry deviation. It is also seen that the spectrum indicates additional distorted regions–two additional peaks at the 21–28 and 81–88 segments (and their counterparts in the second arm of the dimer). The origin of this distortion becomes clear upon careful examination of its 3D structure (Fig 7A): It is seen that the two segments are over-crowdedly very close to each other, and thus, to alleviate this disfavored situation, these segments give-up some of the mutual symmetry for better spatial alignment. It is thus evident that the CSM spectrum and the running ruler method can be used generally for analyzing structural features of proteins other than those originating from the swap mechanism.

Next, let us analyze a case where the hinge peak does exist, but is not the highest, specifically, interleukin-5, Fig 4D, which is a swapped domain protein with a hinge region of 8 amino-acids[15]. Applying the running ruler analysis on this protein creates a CSM spectrum with few peaks (Fig 5D), two of which are higher than the hinge region peak (81–88). The most distortive segment in the structure is at the C-terminal segment (indicated by a blue arrow). Such zones, of either N- or C- terminal segments, tend to distort from perfect symmetry. This is so because of the flexibility of the polypeptide chain termini. This observation is seen again in the C- terminal segment of the N-terminal domain of CD2 (Fig 5A). The second highest peak of interleukin-5 at 38–45 (Fig 5D, by a red arrow) belongs to a segment which is structurally located near the hinge region of the second monomeric sub-unit (Fig 7B). Since the hinges pair region itself is asymmetric, it exerts its distortive influence on neighboring surrounding areas by inter-segment interactions. These neighboring segments are loops, which are flexible areas, thus their distortion surpass that of the hinge-pair areas. The practical conclusion is that if one selects the symmetry analysis tool in order to identify possible hinge areas, then if several peaks appear in the spectrum, visual inspection, as is often practiced in reports on the domain-swapping mechanism, is helpful in eliminating non-relevant segments.

Next, we demonstrate the usefulness of the symmetry analysis, when one wishes to analyze differences in propositions as to hinge identifications by various methods. For example, the reported proposition of Eisenberg[15] for the hinge area location in the TrkA-d4 dimer is the short segment of three amino-acids at positions 297–299. On the other and, Huang et al[19] used Eisenberg's method followed by manual inspection of the structure and proposed that the hinge area is wider and spans over positions 295–299. We have tested these two propositions by producing CSM spectra once with a running ruler of size 3, and once with size 5 (Fig 5E, black dots and red triangles, respectively). With size 3 (according to Eisenberg) the peak appears at 295, that is, the hinge region location is 295–297, a significant shift for such a small hinge region; however, when size 5 is applied (according to Huang) the spectrum indicates the location of the hinge region to be 295–299, in agreement with Huang et al. The fact that size 5 is apparently more relevant than size 3 is also in agreement with our previous analyzed example, drawing attention to the possibility that the distortive effect of the hinge is exerted beyond its minimal suggested size.

We also examined the possibility that the swapped-dimer hinge regions, which are the sites of maximal asymmetry, are also related to maximal flexibility. We therefore generated flexibility spectra for domain swapped structures in Figs 3 and 5 where hinge regions are the sites of maximal asymmetry. The flexibility of each segment in the spectrum was represented by the average atomic displacement factor (ADPs, crystallographic temperature factors) of the atoms in this segment, and the results are shown in S3 Fig. As can be seen, there is no correlation between the CSM spectrum and the flexibility spectrum of each protein. In each spectrum the hinge region is indicated by a local peak, and it is clearly seen that it is not the highest peak. This observation strengthens the interpretations provided by the CSM analysis tool, because it shows that the symmetry distortion of the hinge regions is not a thermal noise phenomenon.

### Probability analysis

In this section we answer the following question: since the identification of the hinge region is based on the assumption that symmetry deviations tend to concentrate in that region, what is the probability that the observed hinge symmetry deviation is more than would be expected from random distribution of asymmetries throughout the protein? For that purpose we resort to the local symmetry analysis, which as explained in the Methods section, evaluates the CSM value of ***C***_***2***_-symmetry related amino-acid pairs (one amino-acid in one monomer, and its counter near ***C***_***2***_-symmetric amino-acid on the second monomer). In a sense, this analysis may also be considered as a "running ruler" analysis with a ruler size of one amino-acid. Here are the details of the statistical probability analysis:

We first run the local symmetry analysis on the whole protein, and get a list of all ***S***(***C***_***2***_***)*** values of all of the amino-acid pairs of the protein; that list is composed of *N* numbers, the number of amino-acids in one monomeric polypeptide chain in the oligomer. That list is arranged in a descending order of the ***S***(***C***_***2***_***)*** values, out of which the first *d*-most distorted pairs are taken, where *d* can be any number smaller than or equal to *N* (*d* ≤ *N*). Next we check how many–*x*—(if any) of these *d*-most distorted pairs appear in the hinge of length *h*. We then evaluate the probability, *P(r)*, that *r* = *x* distorted amino-acid pairs from the *d*-list will appear in a stretch of length *h* within a protein of length *N*. The probability that *at least x* amino-acids are in the hinge must include also the probability to find *r* = *x* + 1 amino acids from the *d*-list, *r* = *x* + 2 amino acids and so on, up to *h* amino-acids from the *d*-list. For our specific application, we find it therefore relevant to take the special case of *d = h*, for which *P(r)* is:

$$P(r)=\frac{\left(\begin{array}{c} h\\ r \end{array})∙(\begin{array}{c} N-h\\ h-r \end{array}\right)}{\left(\begin{array}{c} N\\ h \end{array}\right)}.$$

(See S1 Appendix and S1 Fig for the derivation of this equation). The probability that at least *x* amino-acids appear in the hinge of length *h*-length is then:

$$P=\sum_{r=x}^{r=h}P(r).$$

Applying this calculation we found (Table 2) that in the vast majority of the analyzed proteins, the number of the most distorted amino-acids which reside in the hinge exceeds by far the probability of that to happen, compared to random distribution of these distorted amino-acids in the whole protein. For instance, let us take again the RNase A N-terminal swapped dimer (Fig 3, and PDB code 1A2W in Table 2), which has a hinge region size of *h* = 8 amino-acids. Five amino-acids in the protein are found in its hinge region, and thus *x* = 5. The calculated probability of that to happen coincidentally in a protein of 124 amino-acids (the size of each subunit) is 0.001%. It should be noted that the condition *d = h* is quite stringent, because it may well be that the symmetry deviation of *d* > *h* amino acids is considerable as well, and in that case the, the chances of having a symmetry-distorted amino-acid in the hinge, increases. Let us check for example *d* = 2 ∙ *h* for the same RNase A N-terminal swapped dimer (*h* = 8). Increasing d to be 16 (2 ∙ *h*) changes the list of the most distorted amino acids to: 85, 22, 20, 101, 17, 98, 21, 19, 18, 100, 81, 16, 23, 99, 28, 31. This means that now all the amino acids in the hinge region (underlined) are in the list of the most distorted amino acids. The probability of that to happen coincidentally is 1 ∙ 10^−6^%, namely three orders of magnitude less than the probability presented above.

**Table 2:** Probability analysis of symmetry distortion at the hinge range.

PDB ID	Polypeptide chain length	Hinge region	Most distorted amino-acids^(a)^	Quantity of most distorted amino-acids in hinge region	Probability of the observation
1BSR	124	17–24	20, 34, 37, 17, 19, 22, 18, 24	6 / 8	5*10^−3^%
1A2W	124	16–23	85, 22, 20, 101, 17, 98, 21, 19	5 / 8	1*10^−3^%
1BLB	174	81–89	86, 107, 87, 88, 127, 169, 89, 148, 84	5 / 9	1*10^−3^%
1L5X	270	245–249	247, 245, 38, 248, 43	3 / 5	3*10^−3^%
1QB3	109	89–95	91, 90, 9, 38, 37, 89, 95	4 / 7	0.02%
1CQZ	479	223–238	94, 233, 135, 231, 91, 90, 188, 232, 223, 93, 427, 204, 430, 420, 63, 228	5 / 16	0.1%
2C5J	82	63–67	66, 63, 6, 64, 69	3 / 5	0.1%
1R8J	264	163–171	170, 259, 167, 271, 238, 169, 232, 231, 278	3 / 9	0.2%
1EN7	157	62–71	67, 71, 89, 68, 93, 87, 70, 28, 96, 74	4 / 10	0.2%
2QYP	78	21–23	23, 21, 19	2 / 3	0.3%
2HKN	72	37–39	38, 36, 37	2 / 3	0.3%
1JS0	124	111–114	113, 64, 112, 69	2 / 4	0.5%
2SPC	107	70–73	1, 3, 73, 71	2 / 4	0.6%
1TIJ	112	57–60	9, 106, 58, 57	2 / 4	0.6%
1CDC	96	43–49	46, 47, 36, 79, 43, 91, 88	3 / 7	0.8%
1AOJ	60	36–41	39, 21, 38, 37, 20, 19	3 / 6	1%
2CN4	173	47–52	39, 49, 74, 51, 63, 88	2 / 6	1%
1WWA	101	295–299	296, 347, 349, 295, 350	2 / 5	2%
2NZ7	93	91–95	39, 101, 91, 95, 35	2 / 5	2%
1WKQ	155	117–123	60, 61, 76, 119, 123, 96, 148	2 / 7	3%
1L5B	101	48–53	49, 37, 52, 1, 40, 32	2 / 6	4%
1SCE	102	86–91	60, 88, 87, 35, 101, 59	2 / 6	4%
1E7D	157	87–101	68, 74, 93, 89, 96, 101, 67, 71, 59, 22, 28, 98, 38, 43, 27	4 / 15	4%
1Q8M	121	21–23	22, 124, 63	1 / 3	7%
1A64	94	42–48	44, 27, 87, 26, 86, 45, 20	2 / 7	8%
1G6U	48	32–33	32, 42	1 / 2	8%
1N9J	98	46–49	2, 48, 1	1 / 3	9%
1FYR	95	122–124	122, 135, 145	/ 3	9%
1OBP	155	121–124	96, 157, 153, 123	1 / 4	10%
1LMK	239	122–126	16, 126, 85, 127, 84	1 / 5	10%
1W5F	316	215–220	288, 284, 300, 306, 308, 217	1 / 6	11%
1R7H	74	49–51	40, 37, 49	1 / 3	12%
5CRO	61	54–56	39, 56, 60	1 / 3	14%
1X0G	104	34–37	36, 94, 93, 98	1 / 4	15%
2OYA	102	428–431	431, 419, 460, 418	1 / 4	15%
2OCT	95	46–49	68, 30, 81, 46	1 / 4	16%
1BH5	177	24–36	28, 179, 183, 27, 77, 110, 143, 13, 95, 16, 157, 96	2 / 13	24%
1J30	137	69–82	22, 80, 15, 41, 47, 138, 134, 135, 108, 139, 120, 51, 3, 69	2 / 14	43.3%
1H8X	126	17–24	125, 17, 31, 66, 91, 100, 37, 67	1 / 8	42%
1HUL	107	81–87	110, 112, 50, 109, 42, 40, 111, 39	0 / 8	(see text)

^(a)^ Amino acids are listed by decreasing order; amino acids in the hinge region are underlined.

See Fig 3, Figs 5 and 6 and S2 Fig. For data sources see ref.'s [38–77].

Returning to Table 2, similar (*d* = *h*) calculations carried out on all of the proteins analyzed above (Fig 3, Figs 5 and 6 and S2 Fig), indicate that the probabilities of having the actual observed concentration of distortion in the hinge area, are all well below 15%. As exceptions are highlighting the rule, we comment on the last entry in the Table, Interleukin-5 (IL-5, PDB code: 1HUL): This protein does not have “most-distorted amino-acids” in the hinge region because another region in the protein is more distorted–see Fig 5D—and yet, as also seen in that figure, applying the running ruler analysis clearly identifies the whole hinge region as a peak in the CSM spectrum.

## Conclusions

In conclusion, in relation to the question of ‘why do oligomers settle for imperfect symmetry if symmetrization is so advantageous’ we have explored here the parameter of the mechanism of the oligomerization. Taking the domain swapping mechanism we have shown that the mechanism of oligomerization is an important parameter in affecting the symmetry of the final oligomer (other key parameters are listed in the Introduction). The structure of protein oligomers is a reflection of their formation, and this is translated into the symmetry distortions. The new way of looking at swapped domain dimeric proteins offered by this study—through symmetry–allows comparative quantification of the effects of that mechanism. This method identifies the hinge regions in those proteins through the symmetry perspective, with no need of structural information on the monomeric form of the non-swapped protein (information that does not always existed). In many cases this symmetry analysis indicates the hinge segments as the major contributor to the symmetry distortions in the protein (it is always a contributor, even if not the major one). We found that in the vast majority of the analyzed proteins, the number of the most distorted amino-acids which reside in the hinge exceeds by far the probability of that to happen, compared to random distribution of these distorted amino-acids in the whole protein. And last but not least, we showed that the CSM spectrum and the running ruler method can be used generally for analyzing structural features of proteins, other than those associated with the hinge region.

## Supporting information

S1 Appendix Further explanation about the hinge symmetry probability analysis. (PDF)

S1 FigVisual explanation of the probability calculation for the question 'what is the probability that *at least x* amino-acids out of the *d* most distorted amino-acids appear in a given *h*-length-segment?'.The assumptions: (a) *N* = 7, namely, a dimeric protein composed of two subunits, each of 7-amino acids (a row of circles). (b) *h* = 3. The length of the hinge region is 3 amino acids and it placed as a sequence of at locations 2,3,4 (indicated by the bar); (c) *d* = *h* = 3. There is a list of the 3 most distorted amino acids (orange circles); (d) The experimental observation is that 2 out of the *d* = 3 most distorted amino-acids are located in the hinge. There are $\left(\begin{array}{c} N\\ d \end{array}\right)=35$ ways of placing the 3 most-distorted amino-acids in the set of 7 amino-acids; in each of these ways, the hinge region contains 0–3 amino acids out of the 3 most distorted amino-acids (*r* = 0,1,2,3).(PDF)

S2 Fig Additional running ruler symmetry analyses (see also Fig 5). The neighborhood of the hinge region is shown. Black arrows—the hinge region. (**a**) scaffold protein IscA (1X0G), (**b**) sulerythrin (PDB code: 1J30), (**c**) Soluble epoxide hydrolase (PDB code: 1CQZ), (**d**) Cyclin-dependent kinase (PDB code: 1QB3), (**e**) Designed helical bundle (PDB code: 1G6U), (**f**) Endonuclease VII (PDB code: 1EN7), (**g**) Guanine deaminase (PDB code: 1WKQ), (**h**) T-SNARE (PDB code: 2C5J), (**i**) Hemophore HasA (PDB code: 2CN4), (**j**) Dynactin-1 (PDB code: 2HKN), (**k**) Caspase-recruitment domain CARD (PDB code: 2NZ7), (**l**) Cystatin B (PDB code: 2OCT), (**m**) Macrophage receptor MARCO (PDB code: 2OYA), (**n**) Saposin C Dimer (PDB code: 2QYP), (**o**) Survival protein E (PDB code: 1L5X), (**p**) Endonuclease VII (PDB code: 1E7D), (**q**) Suc1 (PDB code: 1SCE), (**r**) Cro repressor protein (PDB code: 5CRO). See Table 1 for more information. For data sources see ref.'s [60–77].
(PDF)

S3 Fig Comparison of the CSM running ruler symmetry analysis with the average atomic displacement factor (ADP) flexibility parameter. The black arrows indicate the hinge regions. PDB codes of analyzed proteins: (a) 1A2W, (b) 1CDC, (c) 1A64, (d) 1WWA.
(PDF)
