# Supplementary material for: The enigma of the near-symmetry of proteins: Domain swapping
Source: PLoS One. 2017 Jul 14;12(7):e0180030. doi: 10.1371/journal.pone.0180030 (PMC5510828; doi:10.1371/journal.pone.0180030)
Supplement: S1 Appendix — (PDF) [file pone.0180030.s001.pdf]

The enigma of the near-symmetry of proteins: Domain swapping

Maayan Bonjack-Shterengartz David Avnir

Institute of Chemistry and the Lise Meitner Minerva Center for Computational Quantum Chemistry, The Hebrew University of Jerusalem, Jerusalem 91904, Israel

Supporting Information

S1 Appendix. Further explanation about the hinge symmetry probability analysis

We answer the question 'what is the probability that *at least* x amino-acids out of the d most distorted amino-acids appear in a given h-length-segment?'. In our application, x is the number of most distorted amino acids found experimentally in the hinge region of length h. For that purpose we first answer the question 'what is the probability that *exactly* x pairs out of the d most distorted amino-acids appear in a given h-length-segment?'

Let us demonstrate the answers and the relevant equations with a specific example, taking a dimeric protein, composed of two monomers, each of 7-amino acids ($N = 7$). Let us assume the following: A. The length of the hinge region is 3 amino acids ($h = 3$) and it is placed as a sequence at locations 2,3,4 (S1 Fig, the bar). B. We have a list of the d most distorted amino acids so that $d = h = 3$ (orange in S1 Fig). C. The experimental observation is that $x = 2$ out of the $d = 3$ most distorted amino-acids are located in the hinge; *what is the probability of that to happen?*

There are $\binom{N}{d} = \binom{7}{3} = 35$ ways of placing the 3 most-distorted amino-acids in the set of $N = 7$ amino-acids, all of which are shown in Fig. S1. In each of these 35 ways there appear in the hinge region 0-3 amino acids out of the 3 most distorted amino-acids ($r = 0,1, \ldots, h$). What then is the probability that *exactly* x ($r = 2$) amino acids out of the 3 most distorted amino-acids will appear in the hinge? Direct counting of these cases in the figure show that there are 12 possibilities of having two oranges in the hinge, obeying:

(1) $$\binom{h}{x} \cdot \binom{N-h}{d-x}$$

Here, $\binom{h}{x}$ is the number of ways of placing x most-distorted amino-acids in the hinge region, and $\binom{N-h}{d-x}$ is the number of ways of placing the d-x most-distorted amino-acids outside of the hinge region.

Therefore the probability of that to happen is $\frac{12}{35} = 34\%$, obeying

(2) $$P(r) = \frac{\binom{h}{r} \cdot \binom{N-h}{d-r}}{\binom{N}{d}}$$

Our next step is to ask 'what is the probability that *at least* 2 amino-acids out of the 3 most distorted amino-acids will in the hinge?'. In our example, only $x = 3$ is relevant, and there is only one additional option for that (top line in the figure). That number is obtained from eq. 1, and its probability 3% is obtained from eq. 2. Therefore the answer for the "at least" question is a probability of 37%, obtained from

(3) $$P = \sum_{r=x}^{r=h} P(r)$$
