# Supplementary material for: The enigma of the near-symmetry of proteins: Domain swapping
Source: PLoS One. 2017 Jul 14;12(7):e0180030. doi: 10.1371/journal.pone.0180030 (PMC5510828; doi:10.1371/journal.pone.0180030)
Supplement: S1 Fig — The assumptions: (a) N = 7, namely, a dimeric protein composed of two subunits, each of 7-amino acids (a row of circles). (b) h = 3. The length of the hinge region is 3 amino acids and it placed as a sequence of at locations 2,3,4 (indicated by the bar); (c) d = h = 3. There is a list of the 3 most distorted amino acids (orange circles); (d) The experimental observation is that 2 out of the d = 3 most distorted amino-acids are located in the hinge. There are (Nd)=35 ways of placing the 3 most-distorted amino-acids in the set of 7 amino-acids; in each of these ways, the hinge region contains 0–3 amino acids out of the 3 most distorted amino-acids (r = 0,1,2,3). (PDF) [file pone.0180030.s002.pdf]

The enigma of the near-symmetry of proteins: Domain swapping

Maayan Bonjack-Shterengartz David Avnir

Institute of Chemistry and the Lise Meitner Minerva Center for Computational Quantum Chemistry, The Hebrew University of Jerusalem, Jerusalem 91904, Israel

Supporting Information

S1 Fig. **Visual explanation of the probability calculation for the question 'what is the probability that *at least x* amino-acids out of the *d* most distorted amino-acids appear in a given *h*-length-segment?'.** The assumptions: (a) $N = 7$, namely, a dimeric protein composed of two subunits, each of 7-amino acids (a row of circles). (b) $h = 3$. The length of the hinge region is 3 amino acids and it placed as a sequence of at locations 2,3,4 (indicated by the bar); (c) $d = h = 3$. There is a list of the 3 most distorted amino acids (orange circles); (d) The experimental observation is that 2 out of the $d = 3$ most distorted amino-acids are located in the hinge. There are $\binom{N}{d} = 35$ ways of placing the 3 most-distorted amino-acids in the set of 7 amino-acids; in each of these ways, the hinge region contains 0-3 amino acids out of the 3 most distorted amino-acids ($r = 0,1,2,3$).

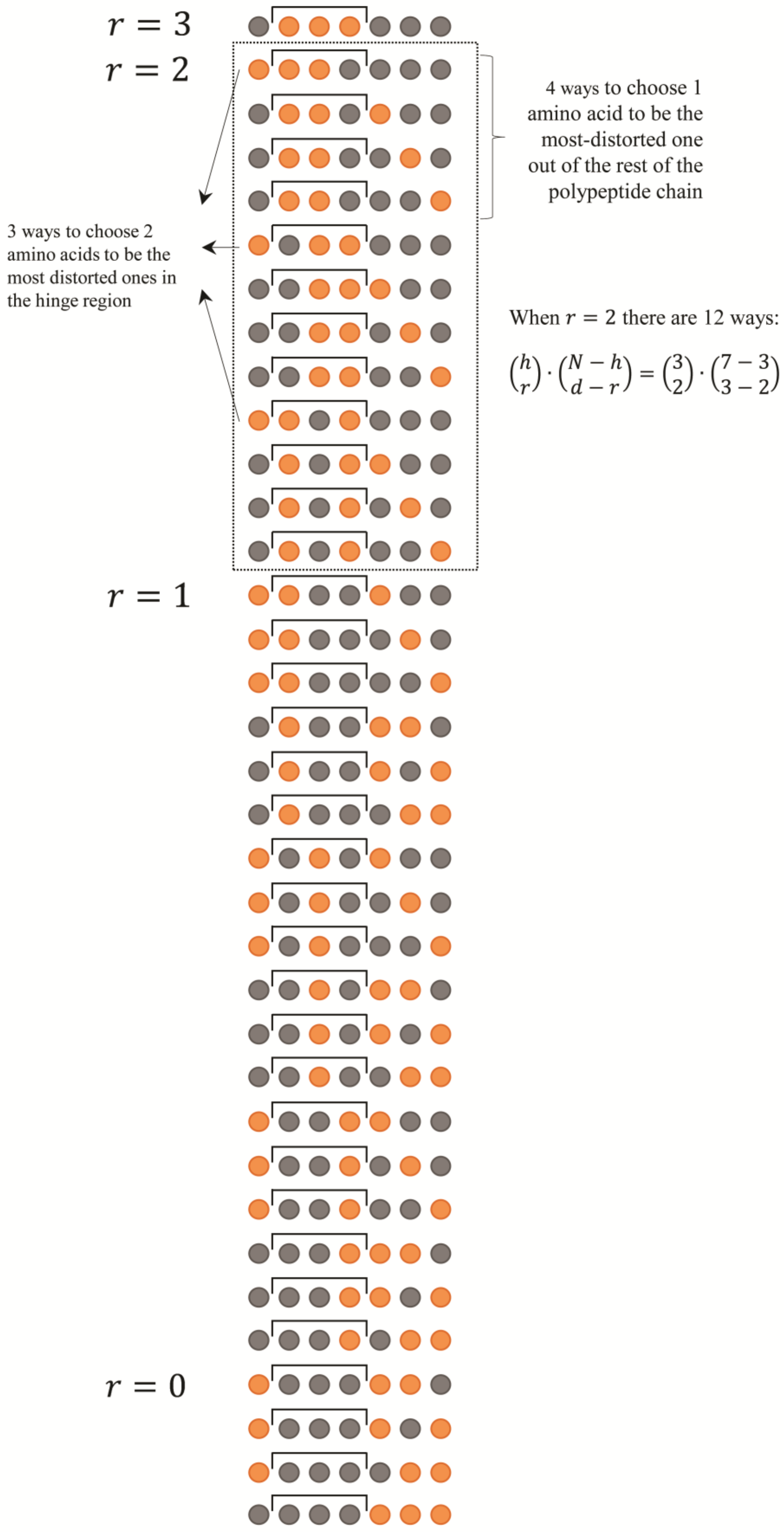

$r = 3$
$r = 2$
4 ways to choose 1 amino acid to be the most-distorted one out of the rest of the polypeptide chain
3 ways to choose 2 amino acids to be the most distorted ones in the hinge region
When $r = 2$ there are 12 ways:
$\binom{h}{r} \cdot \binom{N-h}{d-r} = \binom{3}{2} \cdot \binom{7-3}{3-2}$
$r = 1$
$r = 0$
