# Supplementary material for: The enigma of the near-symmetry of proteins: Domain swapping
Source: PLoS One. 2017 Jul 14;12(7):e0180030. doi: 10.1371/journal.pone.0180030 (PMC5510828; doi:10.1371/journal.pone.0180030)
Supplement: S2 Fig — The neighborhood of the hinge region is shown. Black arrows—the hinge region. (a) scaffold protein IscA (1X0G), (b) sulerythrin (PDB code: 1J30), (c) Soluble epoxide hydrolase (PDB code: 1CQZ), (d) Cyclin-dependent kinase (PDB code: 1QB3), (e) Designed helical bundle (PDB code: 1G6U), (f) Endonuclease VII (PDB code: 1EN7), (g) Guanine deaminase (PDB code: 1WKQ), (h) T-SNARE (PDB code: 2C5J), (i) Hemophore HasA (PDB code: 2CN4), (j) Dynactin-1 (PDB code: 2HKN), (k) Caspase-recruitment domain CARD (PDB code: 2NZ7), (l) Cystatin B (PDB code: 2OCT), (m) Macrophage receptor MARCO (PDB code: 2OYA), (n) Saposin C Dimer (PDB code: 2QYP), (o) Survival protein E (PDB code: 1L5X), (p) Endonuclease VII (PDB code: 1E7D), (q) Suc1 (PDB code: 1SCE), (r) Cro repressor protein (PDB code: 5CRO). See Table 1 for more information. For data sources see ref.'s [60–77]. (PDF) [file pone.0180030.s003.pdf]

The enigma of the near-symmetry of proteins: Domain swapping

Maayan Bonjack-Shterengartz David Avnir

Institute of Chemistry and the Lise Meitner Minerva Center for Computational Quantum Chemistry, The Hebrew University of Jerusalem, Jerusalem 91904, Israel

Supporting Information

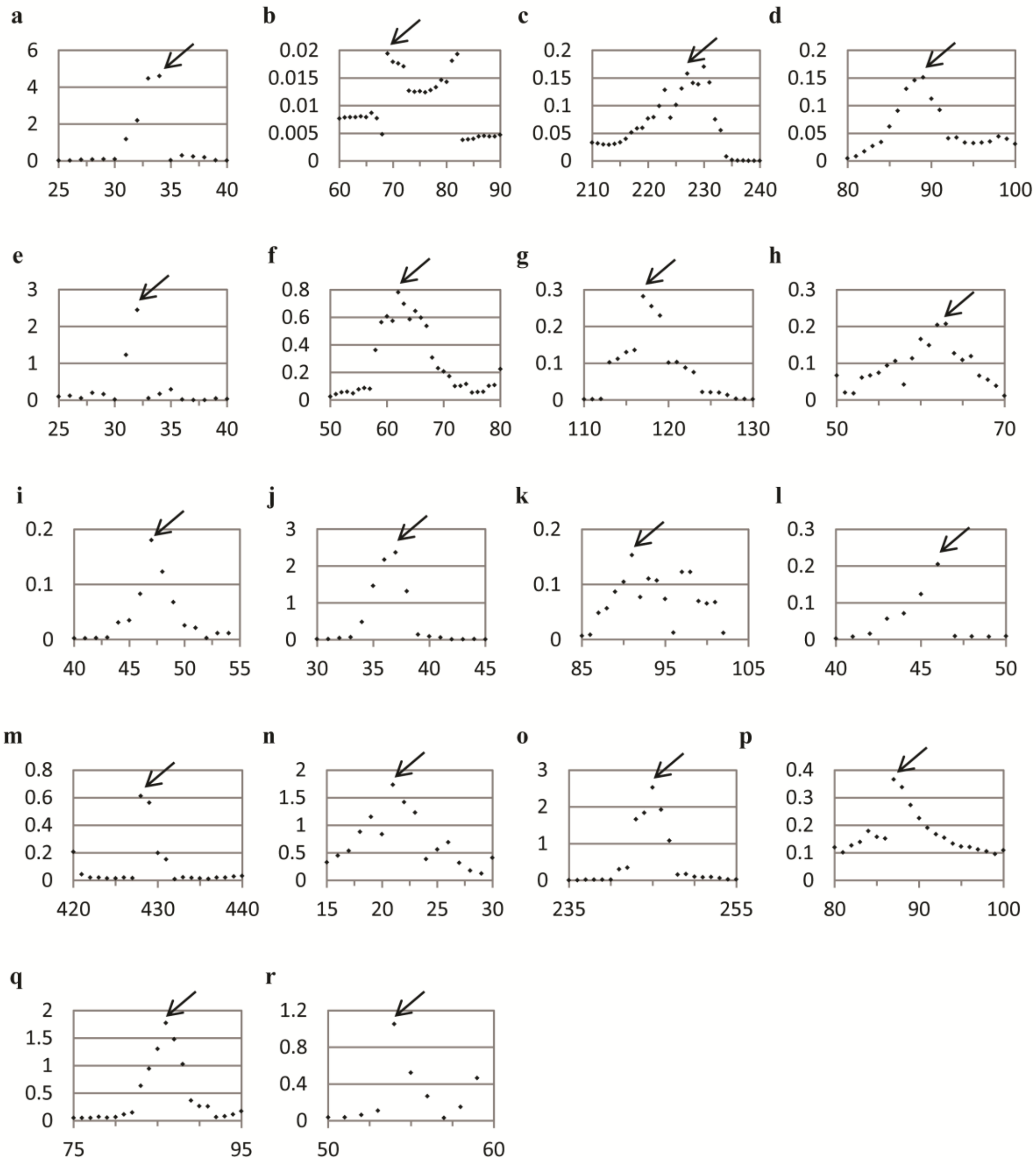

S2 Fig. Additional running ruler symmetry analyses (see also Fig. 5). The neighborhood of the hinge region is shown. Black arrows - the hinge region. (**a**) scaffold protein IscA (1X0G), (**b**) sulerythrin (PDB code: 1J30), (**c**) Soluble epoxide hydrolase (PDB code: 1CQZ), (**d**) Cyclin-dependent kinase (PDB code: 1QB3), (**e**) Designed helical bundle (PDB code: 1G6U), (**f**) Endonuclease VII (PDB code: 1EN7), (**g**) Guanine deaminase (PDB code: 1WKQ), (**h**) T-SNARE (PDB code: 2C5J), (**i**) Hemophore HasA (PDB code: 2CN4), (**j**) Dynactin-1 (PDB code: 2HKN), (**k**) Caspase-recruitment domain CARD (PDB code: 2NZ7), (**l**) Cystatin B (PDB code: 2OCT), (**m**) Macrophage receptor MARCO (PDB code: 2OYA), (**n**) Saposin C Dimer (PDB code: 2QYP), (**o**) Survival protein E (PDB code: 1L5X), (**p**) Endonuclease VII (PDB code: 1E7D), (**q**) Suc1 (PDB code: 1SCE), (**r**) Cro repressor protein (PDB code: 5CRO). See Table 1 for more information. For data sources see ref.'s 60–77.
