# Supplementary material for: The enigma of the near-symmetry of proteins: Domain swapping
Source: PLoS One. 2017 Jul 14;12(7):e0180030. doi: 10.1371/journal.pone.0180030 (PMC5510828; doi:10.1371/journal.pone.0180030)
Supplement: S3 Fig — The black arrows indicate the hinge regions. PDB codes of analyzed proteins: (a) 1A2W, (b) 1CDC, (c) 1A64, (d) 1WWA. (PDF) [file pone.0180030.s004.pdf]

The enigma of the near-symmetry of proteins: Domain swapping

Maayan Bonjack-Shterengartz David Avnir

Institute of Chemistry and the Lise Meitner Minerva Center for Computational Quantum Chemistry, The Hebrew University of Jerusalem, Jerusalem 91904, Israel

Supporting Information

a

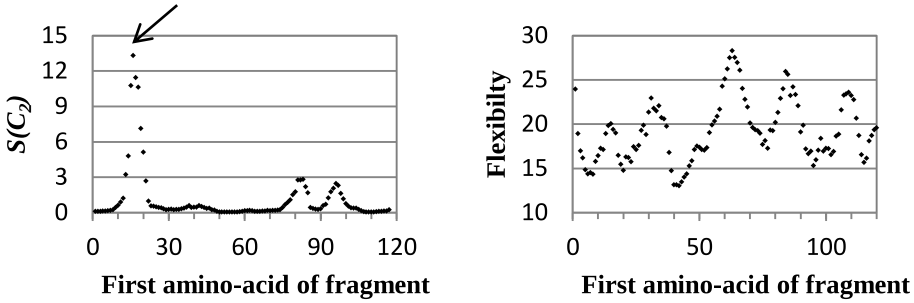

b

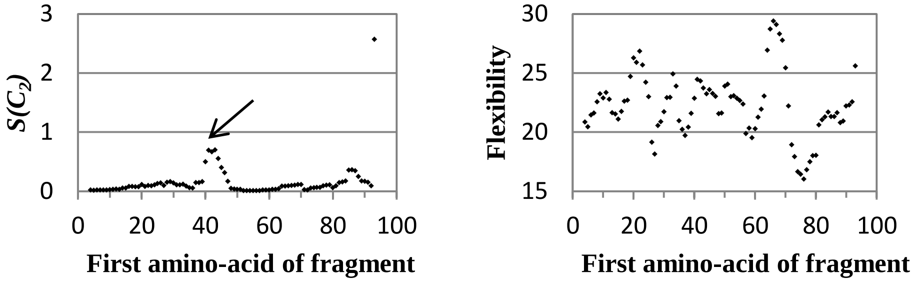

c

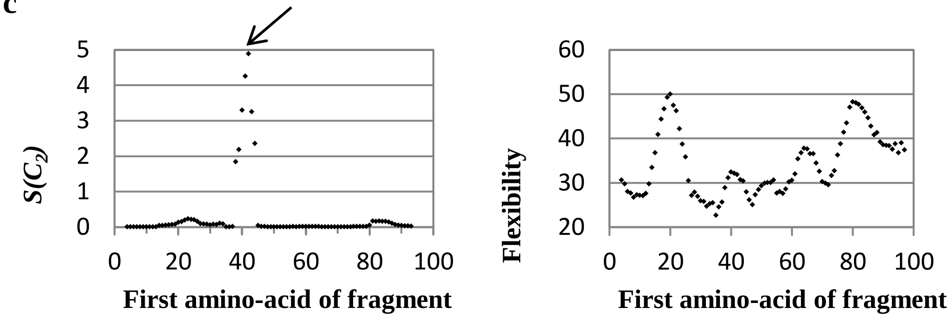

d

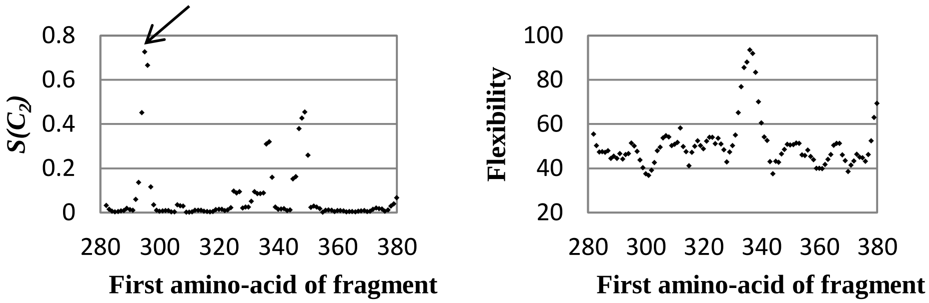

S3 Fig. Comparison of the CSM running ruler symmetry analysis with the average atomic displacement factor (ADP) flexibility parameter. The black arrows indicate the hinge regions. PDB codes of analyzed proteins: (a) 1A2W, (b) 1CDC, (c) 1A64, (d) 1WWA.
